# Lightweight Artificial Intelligence for Secure Data Communication in Energy-Constrained Healthcare Devices

**DOI:** 10.1155/2022/7934582

**Published:** 2022-08-31

**Authors:** Suyog Vinayak Pande, K. Nattarkannan, M. Rama Bai, Dhiraj Kapila, P. Anandan, Nilamadhab Mishra, Worku Abera

**Affiliations:** ^1^Department of Electronics and Telecommunication Engineering, Mukesh Patel School of Technology Management and Engineering, Shirpur Campus, Mumbai, Maharashtra, India; ^2^Department of Artificial Intelligence and Machine Learning, Saveetha School of Engineering, Saveetha Institute of Medical and Technical Sciences(SIMATS), Chennai, Tamil Nadu, India; ^3^Department of Computer Science and Engineering, Mahatma Gandhi Institute of Technology, Ranga Reddy Dist, Hyderabad 500075, Telangana, India; ^4^Department of Computer Science & Engineering, Lovely Professional University, Phagwara, India; ^5^School of Computer Science and Engineering, Vellore Institute of Technology, Chennai 600127, India; ^6^School of Computing Science and Engineering, VIT Bhopal University, Bhopal, Madhya Pradesh, India; ^7^Department of Food Process Engineering, College of Engineering and Technology, Wolkite University, Wolkite, Ethiopia

## Abstract

Logistics is the transfer of goods from one place to another, mostly from the production house to the customers. A logistics network is a set of operations that involve designing, production, and marketing the goods. Cold-chain logistics are those that needed to be transported in a cold refrigeration right from the production house to the customer. A secured networking model is essential to handle the logistics networks. In this article, we are going to see an intelligent secured networking model to identify the optimal path for cold-chain logistics to hospitals. The optimal pathfinder is used to find the path between point A to point B, which is short and best. It also considers the road traffic and cost of transport. The cold-chain logistics to the hospitals include medicines and vaccines, which are to be stored at a particular temperature. Thus, path optimization is more essential in cold-chain logistics to hospitals than other types of logistics. In this research, the bee-ant optimization algorithm (BAOA) is proposed to perform the intelligent transportation to the hospitals. The proposed algorithm is compared with the existing ant colony optimization (ACO), bee colony optimization (BCO), and neural network model. From the results, it can be observed that the proposed algorithm shows 98.83% for the accurate delivery of logistics to the hospitals.

## 1. Introduction

While analyzing the cold-chain improvement in China, it focuses on three particular fields: commerce electronics, food manufacturing industries, and shopping malls. Here, the author of Ref. [1] had a look at terminal logistics serving centers that are insufficient locally. To reduce the insufficiency, he developed different schemes to manage those logistics spaces. In Ref. [2], the author brought a new idea to implement cloud computing and big data methods to improve fresh food management. This algorithm would also increase the traffic control under transportation and analyze the time required by a refrigerated vehicle with the help of parallel programming. Income management is one of the everyday things for every software company. In that case, cloud computing may cause a tremendous technological change in hardware and software companies. To identify the multiple types of data that can be collected and anatomized by interpreters across the cold chain, the ICT structure is needed to enable data capture and how to use the data for decision-making in cold-chain logistics. Data capture involves several types of data, which corresponds to products and processes. Most systems aim at collecting temperature or ambient-related data [3].

## 2. Literature Review

By the 1950s, VR (virtual reality) had been introduced to this world and by 2021 had developed the growth of graphics, programming, computation technology, and other software programs. Reference [4] demonstrates the actual process of virtual reality and also analyzes its value under cold-chain logistics. Food management is one of the significant recovering fields normally. Every food has a limited period to complete its life cycle. Within the end of its lifetime, it should sell it out to any customers. The authors in Ref. [5] have provided a process for the involvement of cold-chain logistics in all other fields. With the help of cold-chain logistics, there are some possibilities to check the quality of the product. Using such technology makes it possible to scan a product to identify its quality. They are following that Ref. [6] brought us a massive algorithm with an accuracy statement that explains the use of cold-chain logistics after implementing quality control. By presenting a navigating model, the author has described its purpose under the rise of sensor management, processing, and analyzing the data stored and merging it with real-time applications. Here, the dynamic programming algorithm is shown with its results by improving the run time and accuracy, finally demonstrating the output short-range ship management and wave-field problems have been rectified [7].

While traveling, people face different problems on their roads; this will not affect the ordinary traveler. When the same problem is confronted by a delivery truck driver or any other driver, it will affect their work. According to Ref. [8], the author has explained an algorithm named Dijkstra-GA, which is used under the path distribution from a cold-chain logistics. Using IoT (Internet of Things) creates a platform that connects several devices under the same platform. In that case, once we connect the devices under an application or a network, the connections between devices are made more robust, which automatically increases the controllability. This would help to make further operations with the help of cloud-based development [9]. There are different sections in the level of transportation; for example, if a vaccine is to be transported from one country to another country, then under the shifting place and preserve the vaccine, they would take different actions. This kind of activity is cold-chain equipment; through Ref. [10], the author gave us an enormous explanation of the cold chain and its improvement in China. In recent days, artificial intelligence has gained much value, so by utilizing it, technical engineers are trying to create automated things even for business management. If such things happened, robotic process automation would result in a robust and better decision-making platform. This article represents the clarity under key distributions of particular literature and its benefits [11]. By the development of technology, it automates the level of studies for the children, in that way here the author has a detailed architecture of logistics Internet of Things (IoT); after crossing over hundreds of articles, he came to a conclusion about logistics and started to classify it according to potential directions [12]. Finally, there is an actual relationship between the Internet of Things, logistics cloud chain, and technological developments. As a collaboration with several authors, the final paper has been started with different concepts such as virtual reality, machine learning, artificial intelligence (AI), and at last related to the logistics cloud chain with the future results [13]. The main problem in the electric vehicle routing problem will be carrying the products in spite of the heavy traffic, and the introduction of the new ant colony algorithm will help in the same distribution of the vehicles in the congestion period given the correct arrangement of all the products in a cost-effective way [14, 15].

## 3. Motivation of the Study

Cold chain logistics is the safe repository for managing of temperature-sensitive goods from one place of manufacture to the point of consumption. In this process, vehicles but rather warehouses equipped with cooling systems were used. With the support of cold-chain logistics, foods such as processed food, medicine, blood, eye, and kidney are safely transported to a consumer. People will investigate the path optimization technique of cold-chain logistics vehicles based on the improved evolutionary algorithms in this scientific report. The cold-chain logistics vehicle's plan is important to reaching consumers in less time, distance, and cost. Routes are chosen based on data from road transportation. Path optimization algorithms are used for this. The bee-ant optimization algorithm (BAOA) is a type of artificial swarm intelligent technology that is based on bee and bee colony behavior. The cold-chain logistics path is optimized only with the aid of digital technology but instead Global Positioning System (GPS) technology. For the transportation of temperature-sensitive goods, the best short route is discovered. The primary objective of this research would be to shorten the duration, distance, and cost associated with transportation.

## 4. Proposed Model

**Figure 1:**
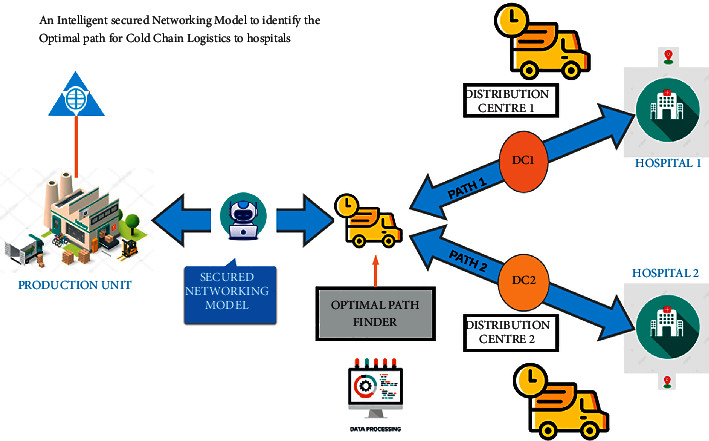
Proposed model for cold-chain logistics to hospitals.

Networking models are created beforehand to ensure the smooth flow in the logistics movement that performs the best as represented in Figure 1. The networking models are used to find the three most important factors. They are as follows:

- Functionality: the sum or any aspect of what a product, similar to a software operation or computing device, can do for a user.
- Cost involved: product costs can include a variety of charges, similar to labor, raw stuff, consumable manufacturing inventories, and general outflow. Total product costs can be determined by adding together the total direct stuff and labor costs as well as the total manufacturing outflow costs.
- Customer satisfaction: how happy clients are with a company's products, services, and capabilities is determined. Customer satisfaction information, including inspections and conditions, can help a company determine how to enhance or changes its products and services.

Before implementing a logistic network, these factors are to be considered to create an efficient, cost-effective way of logistics. There are various types of modeling techniques that are used to deliver the best customer service with good profit at the same time with reduced cost of the logistics handling. In this research, optimization modeling to arrive at an intelligent secured networking model to identify the optimal path for cold-chain logistics to hospitals is considered for evaluation.

The optimization method makes use of mathematical formulas to find a better solution in the cold-chain logistics for hospitals. This method is based on assumed data:

- Number of production units: a process, line, system, conditioning, or approach, or a combination or series, therefore, are used to produce a product (or family of products). A production unit is not the process or the product. It is the combination of the process and the products produced by that process.
- Number of distribution centers: A distribution center is a product storehouse and shipping structure that stores goods a company produces. Distribution centers are a crucial part of the distribution chain for products, order fulfilment, and storing produced goods previous to their payload to wholesale, retail, or clients.
- Inventories to be maintained: effective inventory management lies nearly between these two axes. While it requires further work and planning to achieve an effective operation process, your gains will reflect your effort.
- High-level customer satisfaction: by surpassing their introductory expectations, you will achieve an advanced position of client satisfaction. For example, delivering a remarkably fast and super-friendly and helpful service is one way to exceed basic prospects. Another is to give products and services, which do further than your clients hope.

Linear programming is one of the most used types of optimization model in logistics networks. This method involved optimized use of the resources to arrive at maximum profits. Artificially intelligent computer systems are used to carry out the linear programming method to deliver the best result. This method is very much useful to meet the supply and demand between the two ends. Thus, there is a proper flow of goods from the production units, distribution centers, and hospitals. Thus, path optimization is more essential in cold-chain logistics for hospitals.

Medicines and their related equipment have to maintain a certain room temperature to function properly. In this regard, the production units, distribution centers, inventories, and also transportation systems should be equipped with the required specifications. It is easier to monitor the temperature when the product is in static case similar to the first three locations. However, the process is highly challenging during transportation. To overcome this challenge, cold chain logistics concepts are implemented to monitor the temperature on the move; additionally, bee-ant optimized algorithm is implemented to find the optimal path for transportation. In the proposed algorithm, the bee optimized algorithm is implemented in the forward pass and the ant optimized algorithm in the backward pass. During the forward pass, the optimal pathfinder has to do only the process of finding the path and it should be quicker. The reason the bee optimized algorithm is that the bee has the property to find the shortest path to the food resource from the location of the hive, whereas, during the backward pass, transportation of the material is considered in concern to the logistics. Ant optimization is chosen, as the ant generates the chemical compound named pheromone and it has the property of evaporation. This concept can be interlinked with the cold-chain property of monitoring temperature. When there is a change in the temperature during the transportation, as an alternative arrangement, another logistics can be chosen to continue the process. Hence, the combined model of the bee-ant optimized algorithm is proposed.

## 5. Proposed Work

The optimization model improving supply network efficiency and performance to give customers what they want, when and where they want it while making the business profitable and sustainable appears to take some influencing effects into consideration to represent the real situation, including time-varying transportation, online traffic, traffic levels, customer time consumed, sleekness of innovative products, but it also gives a pathway to charge delay time. The bee-ant optimization algorithm was created to help with the electric vehicle routing for logistics vehicles (EVRP).

It was inspired by the natural rustling actions of honey bees, to find the optimal result. The algorithm performs both an exploitative neighborhood search combined with an arbitrary explorative search. The bee-ant optimization algorithm has been successfully applied to several optimization problems such as multiobjective optimization, neural network training, manufacturing cell arrangement, job shop scheduling for a machine, data clustering, optimizing the design of mechanical factors, image analysis, and supply chain optimization. According to the simulation results, the proposed scheme can effectively prevent congestion problems during the delivery process, lower total distribution costs, and improve the efficiency of a cold-chain logistic distribution system with new items.

The set of all logistics nodes in the network is denoted by *K* = *P* ∪ *M*{0}. The letter *P* stands for the collection of customer touch points. The processing facility is denoted by the number 0; *M* denotes a collection of fast chargers, whereas *K* denotes a collection of all endpoints in a logistics system. H is the set of time periods that occur throughout the day, *H* = {*H*_1_, *H*_2_,……, *H*_*n*_}; *n* is the number of time intervals, G denotes the collection of road segments in the road infrastructure, *G* = {*G*_1_, *G*_2_,……, *G*_*n*_}; *G*  denotes the number of road section types. *D* = {*D*_1_, *D*_2_,……, *D*_*n*_}; *D* is the number of area types in the area set.

Let *ρ*_*i*_ signify the required to charge stations able to charge equipment utilization ratio  *ρ*_*i*_=(*λ*_*i*_/*x*_*i*_*μ*_*i*_) According to the conventional slight equation, the waiting period for such *g*^*th*^SK and trying to charge point *i* is represented by the following equation:

(1) $$\begin{array}{c} h_{ig}^{G}=\sum_{i=1}^{x}\frac{{\left(x_{i}\rho_{i}\right)}^{s_{i}}\rho_{i}}{\lambda_{i}x_{i}!{\left(1-\rho_{i}\right)}^{2}\varphi_{D_{i}}}.{\left(\sum_{n=0}^{x_{i}-1}\frac{{\left(x_{i}\rho_{i}\right)}^{n}}{n!}+\frac{{\left(x_{i}\rho_{i}\right)}^{x_{i}}}{s_{i}!\left(1-\rho_{i}\right)}\right)}^{-1}H_{ig}\times g^{th}\text{SK}. \end{array}$$

In the charging method, the load voltage for the *g*^*th*^SK at endpoint *i* is as shown in the following equation:

(2) $$\begin{array}{c} h_{ig}^{G}=\sum_{i=1}^{g}\frac{S_{\max}-S_{ig}^{H}}{n_{h}}. \end{array}$$

Electric energy consumption is influenced not just by the vehicle's disposition, but by its loads and speeds. Whenever an *SK* with a charge travels at *A*_*k*_ transportation distance *ŋ* km/h *k* on a flat road, the ability to run power *D*(*A*_*k*_, *k*) is represented by the following equation:

(3) $$\begin{array}{c} D\left(A_{k},k\right)=\frac{\left(\left(y+A_{k}\right).g.\int k+\left(\left(R_{n}.Z_{i}.k^{3}\right)/\left(22.56\right)\right)\right)}{\left(3700ŋkm/hk\right)}. \end{array}$$

The genuine speed of such a vehicle in a sufficiently short span of time is used to speed proportional with the determined period of time. The following equation is updated to explain variation in the existing roads through which the driver has planned to travel:

(4) $$\begin{array}{c} k_{ij}\left(t\right)=\overset{j}{\underset{i=1}{\sum }}\left\{k_{ij}^{1},t\in H_{1},k_{ij}^{2},t\in H_{2},k_{ij}^{n},t\in H_{n},\left(i,j\right)\in G.\right. \end{array}$$

In *x*, the allocation of velocity fee on the street does now no longer alter in an identical timeframe (*i*, *j*). *M* range of things are considered, together with avenue type, delivery time period, however additionally *M*_1_, *M*_2_,……, *M*_*n*_ time-various velocity of the car together with such location and additionally transition as with inside the following equation:

(5) $$\begin{array}{c} M=\sum \left\{M_{1},M_{2},\ldots \ldots ,M_{n}\right\}. \end{array}$$

The transmitter and *g*_*ij*_ the receiver need to be one of a kind from the alternative vehicles, and the operator can certainly be clearly connected (WSN with AI) to the desired tool without dispute. Transition pace is $n_{i}-\underline{n}$, which is classed into on-the-spot transition and time change. Space-time transformation, inclusive of the preceding MH, takes a while to comply with the series, which is represented in the following equation:

(6) $$\begin{array}{c} \text{MH}=k_{ij}\left(t\right)\sum_{i=1}^{x}\frac{x\sum_{i=1}^{x}\sum_{j=1}^{x}+\sum g_{ij}\left(n_{i}-\underline{n}\right)\left(n_{j}-\underline{n}\right)}{\sum_{i=1}^{x}\sum_{j=1}^{x}+\sum g_{ij}{\left(n_{i}-\underline{n}\right)}^{2}}. \end{array}$$

The following equation represents the cold-chain logistics vehicles primarily based on totally extrude within the transition time:

(7) $$\begin{array}{c} \text{MH}=k_{ij}\left(t\right)\sum \frac{x\sum_{i=1}^{x}\sum_{j\neq 1}^{x}+\sum g_{ij}\left(n_{i}-\underline{n}\right)\left(n_{j}-\underline{n}\right)}{K^{2}\sum_{i=1}^{x}\times \sum_{j=1}^{x}g_{ij}}. \end{array}$$

The following equation then deals with the calculation of the number of storage media to modify the transaction depending on the time of the cold-chain logistics:

(8) $$\begin{array}{c} k_{ij}\left(t\right)=\sum_{i=1}^{j}\frac{\sum_{j=1}^{q}\sum_{d=1}^{q}\sum_{u=1}^{q}\sum_{y=1}^{x_{d}}\left|s_{ij}-s_{dy}\right|}{2x^{2}t}. \end{array}$$

Multiple sentences related to various *A* vehicle types and data add-ons can indeed be described by vehicle position and conversion, and the number of *s*_*ji*_ − *s*_*dy*_ data can indeed be represented by *t*_*j*_+*t*_*d*_ by the focus arc vehicle speed and duration; according to the following equation, WSN network and AI technology work together as follows:

(9) $$\begin{array}{c} {\text{petri}}_{jd}=\frac{\sum_{A=1}^{d_{j}}\sum_{y=1}^{x_{d}}\left|s_{ji}-s_{dy}\right|}{x_{j}x_{d}\left(t_{j}+t_{d}\right)}. \end{array}$$

A WSN net with code operating procedures based on AI technology is described by equation (10), which provides the *ϑ* direction of the vehicle *ϑ*_*n*_^2^+*ϑ*_*x*_^2^+*B*_1_. *B*_1_ Transformations such as 2*ϑ*_*n*_*ϑ*_*x*_+*B*_1_ correlation between different *τ* objects of a given statement, and 2*τ*_*nx*_+*B*_2_ oriented action sequences have very different types *τ*_*n*_^2^+*τ*_*x*_^2^+*B*_2_ of velocities and times in the declaration:

(10) $$\begin{array}{c} M_{xn}=1-\sum \frac{\left(2\vartheta_{n}\vartheta_{x}+B_{1}\right)\left(2\tau_{nx}+B_{2}\right)}{\left(\vartheta_{n}^{2}+\vartheta_{x}^{2}+B_{1}\right)\left(\tau_{n}^{2}+\tau_{x}^{2}+B_{2}\right)}t\in h_{i}. \end{array}$$

Equation (11) clarifies the modeling method of the influence of the code based on the *M*_*xn*_ network on the optimization performance of the code behavior cold-chain modeling based on the *h*_*i*_ network; the *v*_*q*_ correlation between the *y* network component and the code component is also in accordance with equation (12):

(11) $$\begin{array}{c} K\left(h_{i},g_{j}\right)=\sum K\left(h_{i}\right)K\left(\frac{g_{j}}{h_{i}}\right), \end{array}$$

(12) $$\begin{array}{c} k_{y}=\sum_{y=1}^{y}\frac{2y}{y+1}+\left[\frac{1}{2}+\frac{1}{2y}\right]\sum_{x=1}^{x}{\left[\frac{x_{2}-x_{1}}{3}\right]}^{2}+\sum_{x=1}^{x}\frac{2\left(x_{2}-x_{1}\right)}{3}. \end{array}$$

In order to complete the evolution of *x*_2_ − *x*_1_ from rules to networks, static path analysis techniques are used to analyze and process executable files, as shown in the following equation:

(13) $$\begin{array}{c} P_{jn}=\int_{0}^{\infty }hS_{j}\left(x\right)\int_{0}^{s}\left(x-n\right)\times hS_{n}\left(x\right). \end{array}$$

One program document *x* − *n* provides an associated process *M*_*ih*_ with a certain input object set that has been processed, and the digital output object *P*_*jn*_ that is produced is presented as a result in the following equation:

(14) $$\begin{array}{c} \ln\ln\left(\frac{M_{ih}}{M_{ih}-1}\right)=\sum_{i=0}^{h}\alpha +\beta \text{ln ln}M_{ih}-1+\left(2\vartheta_{n}\vartheta_{s}+B_{1}\right)\left(2\tau_{ns}+B_{2}\right), \end{array}$$

(15) $$\begin{array}{c} \sum_{n=1}^{\theta }Wn*l=\frac{\sum_{n=1}^{\vartheta }\left(T+\left(Y_{i}/\sum_{1}^{x}D_{\varepsilon }\right)/M+Y+YK\right)}{\beta +\alpha \ln\ln M_{in}+\left(K/Y\right)}. \end{array}$$

Equation (15) represents the sum of the unit time of ∑_*n*=1_^*θ*^*Wn∗l* and the total execution time. Execution time includes *β*+*α*lnln*M*_in_+(*K*/*Y*) time spent mainly on roads and client points, and time spent not only on queuing but also on charging points.

## 6. Results and Discussion

**Figure 2:**
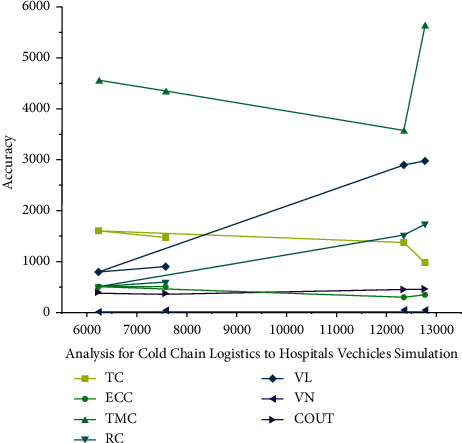
WSN with AI technique performance analysis for cold-chain logistics to hospital vehicle simulation.

Let  *ρ*_*i*_ represent the necessary requirements to charge stations capable of charging equipment capacity utilization ratio *ρ*_*i*_ = (*λ*_*i*_/*x*_*i*_*μ*_*i*_). Its waiting period for these kinds of *g*th *g*^*th*^SK when attempting to start charging point *i* is depicted by equation according to the conventional small equation (1) show in Figure 2 depicts the performance analysis of the Cold-Chain Logistics simulation using WN and AI technologies for intelligent transportation.  Table 1 also provides a detailed numerical representation of this figure. The following metrics are used for evaluation: dataset type, for example, denotes EX-. Total distribution cost is abbreviated as TC. Total travel distance (VD) and ECC (energy consumption and charging) are acronyms for total travel distance and energy consumption and charging, respectively. TMC is the abbreviation for time management cost. RC is the abbreviation for refrigeration cost, VL is the abbreviation for fresh value loss, VN is the abbreviation for vehicle number, and CPUT is the abbreviation for running time (s). In this study, one of four dataset types is chosen at random. In addition, the previously mentioned customer distribution metrics are used for evaluation.

**Figure 3:**
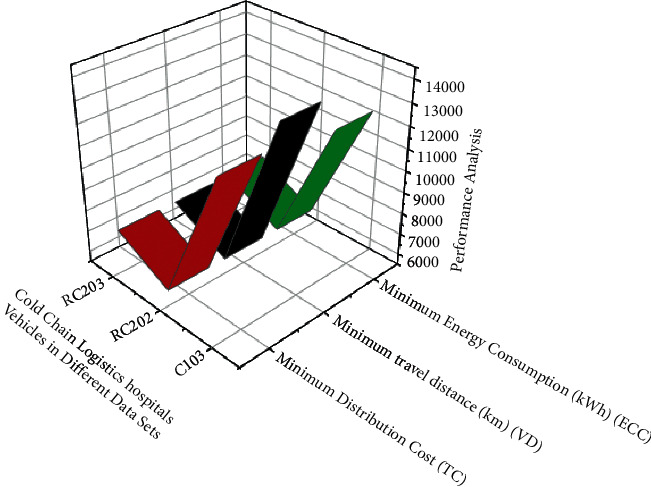
Analysis of cold-chain logistics vehicles in different data sets using WSN and AI techniques.

In *x*, the availability of speed fees on the road no longer changes in the same timeframe (*i*, *j*). *M* a number of factors are taken into account, including avenue type and delivery time frame, but also *M*_1_, *M*_2_,……, *M*_*n*_ time-varying velocity of the car in conjunction with so much location and additionally transformation as with within the following equation (5) to show in Figure 3 depicts data optimization analysis performed on cold-chain logistics vehicles. The optimization method employs the use of intelligent wireless sensor networks for the online ordering of materials, which necessitates the preservation of temperature during resource transportation. This is accomplished through the use of intelligent sensor devices attached to the transporting vehicle to continuously monitor the status of the resources.  Table 2 shows the minimum distribution, travel distance, and energy consumption for transportation.

**Figure 4:**
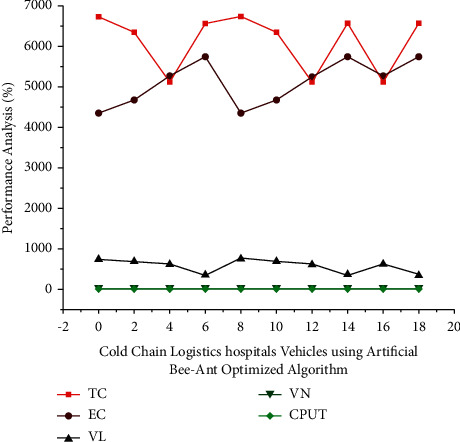
The bee-ant optimized algorithm was used to analyze cold-chain logistics hospital vehicles in various data sets.

One program document  *x* − *n* associates a process *M*_*ih*_ with a specific input item set, which has been filtered, and also the digital signal object *P*_*jn*_, which is produced and preffered as a direct consequence in the process's above equation (14). The performance analysis represented in Figure 4 is carried out in various and varying sizes.  Table 3 shows the quantity of data and the status of the analysis. When the size of the data increases, the corresponding analysis metrics reach their peak values faster than when the data size is small.

**Figure 5:**
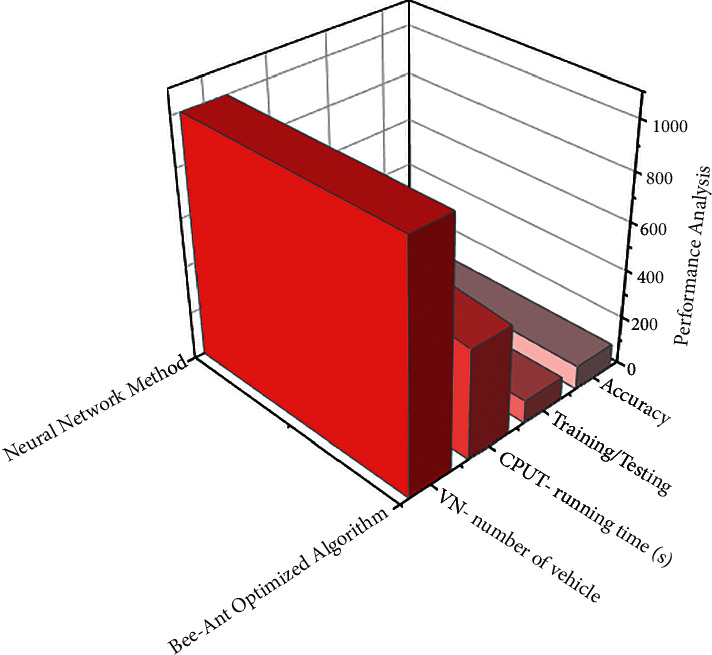
Using the bee-ant optimized algorithm, we analyzed existing systems and cold-chain logistics hospital vehicles in various datasets.

The quantity of the unit of time of ∑_*n*=1_^*θ*^*Wn∗l* and also the maximum completion time are given by Equation (15). The execution time includes *β*+*α*lnln*M*_in_+(*K*/*Y*) time spent primarily on roads but also client points, as well as effort consumed not only having to queue but also charging station charging points are represented (refer to Figure 5). The vehicles used for transportation in cold-chain logistics are equipped with temperature monitoring and many other comparative sensors for constant monitoring of a transporting vehicle. According to Table 4, the number of hospital vehicles considered in the virtual network (VN) and the considered qualify for evaluation are 1000. CPUT represents the time it takes to generate all possible paths and is measured in seconds. Following the selection of the optimal path using artificial intelligence, the system will be able to train in that path but also test the remaining transportation. It has been discovered that the implementation of the ABC algorithm results in an accuracy of 98.83% in optimal path selection but also the transportation of cold logistics along that path.

Cold-chain logistics refers to the safe storage, logistics, and management of temperature-sensitive goods from the point of manufacture to the point of consumption. Vehicles were not used in this process, but rather warehouses outfitted with a cooling system. Foods such as processed foods, medicine, blood, eyes, and kidneys are safely transported to consumers with the help of cold-chain logistics. In this scientific report, researchers will look into the path optimization technique of cold-chain logistics vehicles based on an improved evolutionary algorithm. The plan for the cold-chain logistics vehicle is critical for reaching consumers in less time, distance, and cost. Routes are determined using data from road transportation. The bee-ant optimized algorithm is compared with the results of existing systems (95.54%) and cold-chain logistics hospital vehicles (98.83%) in various datasets. Proposed method provides better results when compared to the existing methods.

## 7. Conclusion

An intelligent transport system has become mandatory in the updated world with the technological evolution. The role of an intelligent system has to focus on all the desired users or clients. The products to be transported should be handled with care with optimal path routing for timely delivery. In recent years, the transportation of logistics has to be concentrated as it involves different medicine that needs certain temperature to be maintained during the transportation. Hence, in this research, cold-chain logistics concepts are proposed to monitor the transportation of medicine with the specified temperature and the bee-ant colony optimization algorithm to transport in the optimal path. From the results, it can be observed that the proposed model has acquired an accuracy performance in optimal path transportation as 98.83%, which is higher when compared to the existing bee colony optimization, ant colony optimization, and the neural network model.

## Figures and Tables

**Table 1:** Analysis of the simulation results for cold-chain logistics hospital vehicles with different consumer distributions.

EX	TC	VD	ECC	TMC	RC	VL	VN	COUT
C103	12783.4	978.5	345.1	5623.6	1745.4	2985.54	9	453.3
C105	12355.2	1359.8	298.6	3547.3	1503.8	2892.93	9	442.9
R202	6245.67	1587.9	487.5	4534.2	492.2	789.32	7	394.4
RC203	7578.54	1467.3	494.3	4325.9	586.5	890.7	8	368.7

**Table 2:** Effectiveness conclusion analysis for cold-chain logistics hospital vehicles in different datasets using WSN and AI techniques.

EX	Minimum distribution cost (TC)			Minimum travel distance (km) (VD)			Minimum energy consumption (kWh) (ECC)		
	TC	VD	ECC	TC	VD	ECC	TC	VD	ECC
C103	12783.4	978.5	345.1	13756.3	987.6	298.1	12234.9	980.2	256.7
R202	6245.67	1587.9	487.5	6234.2	1034.2	502.3	6567.3	1034.7	321.3
RC203	7578.54	1467.3	494.3	7568.3	1325,7	474.1	7645.1	1567.2	399.1

**Table 3:** Cold-chain logistics hospital vehicle performance analysis in different datasets using the bee-ant optimized algorithm in WSN with AI.

Various data	TC	EC	VL	VN	CPUT
10	6734.1	4356.7	752.5	11	5
9	6345.9	4675.2	689.4	9	0
7	5136.1	5258.6	623.2	13	1
5	6567.9	5753.8	356.0	16	1

**Table 4:** Using the bee-ant optimized algorithm, we compared the results of existing systems and cold-chain logistics hospital vehicles in various datasets.

Algorithm	VN-number of vehicles	CPUT-running time (s)	Training/testing (%)	Accuracy (%)
Bee-ant optimized algorithm	1000	453.3	94.89	98.83
Existing method: ant colony optimization	1000	1427.9	1200.9	97.67
Existing method: bee colony optimization	1000	762.87	637.1	96.982
Existing method: neural network method	1000	563.6	89.23	95.54

## Data Availability

The datasets used and/or analyzed during the current study are available from the corresponding author on reasonable request.
